# Lymph Node Ratio-Based Staging System for Gallbladder Cancer With Fewer Than Six Lymph Nodes Examined

**DOI:** 10.3389/fonc.2020.542005

**Published:** 2020-09-25

**Authors:** Jinjun Li, Yao Sun, Bingqing Zhao, Chuangang Tang, Dongxu Fan, Wenli Jiang, Youlutuziayi Rixiati

**Affiliations:** ^1^Department of Surgery, Tianjin Second People’s Hospital, Tianjin, China; ^2^Vascular Surgery Department, First Affiliated Hospital of Jiamusi University, Jiamusi, China; ^3^Department of Breast Surgery, Xuzhou Central Hospital, The Affiliated Xuzhou Hospital of Medical College of Southeast University, Xuzhou, China; ^4^Department of Biochemistry and Molecular Biology, College of Basic Medical, Navy Medical University, Shanghai, China; ^5^Department of Pathology, Soochow University Medical School, Suzhou, China

**Keywords:** lymph node ratio, gallbladder cancer, staging, SEER, AJCC

## Abstract

**Purpose:**

The 8th edition of the American Joint Committee on Cancer (AJCC) tumor-lymph node-metastasis (TNM) staging system for gallbladder cancer (GBC) recommended that at least six lymph nodes (LNs) should be examined. But most patients with GBC had fewer than six LNs resected. This study aimed to establish an alternative index for assessing the LN status during the staging system for GBC patients with fewer than six LNs retrieved.

**Patients and Methods:**

Patient data was extracted from the Surveillance, Epidemiology, and End Results (SEER) database (cases between 2004 and 2013). X-tile software was used to determine the optimal cutoff value for lymph node ratio (LNR) and a concordance index (C-index) was used to evaluate the discriminatory powers of the two staging systems.

**Results:**

The majority of GBC patients in our cohort (1353, 78.5%) had fewer than six LNs examined. Among patients with inadequate LN examination, the higher number of LNs examined correlated with a lower proportion of patients. Using the TNM staging system, the C-index for patients with fewer than six LNs and patients with six or more LNs screened were 0.636 and 0.704, respectively. Using the staging system based on LNR (TNrM), the C-index for patients with fewer than six LNs retrieved and patients with six or more LNs retrieved were 0.649 and 0.694, respectively. Similar results were observed in patients with gallbladder adenocarcinoma (GBA).

**Conclusion:**

TNrM might be superior to the 8th AJCC TNM staging system for stratifying GBC patients with fewer than six LNs examined, and it can complement TNM for more accurate risk stratification. Future prospective studies are needed to validate our findings.

## Introduction

Gallbladder cancer (GBC) is a relatively rare but lethal malignancy of the biliary tract with a 5-year survival rate of less than 5% (1, 2). The GBC patients are routinely stratified into different risk groups based on the American Joint Committee on Cancer (AJCC) tumor-lymph node-metastasis (TNM) classification. The 8th edition of the AJCC staging system that was updated in 2018 used an altered definition of N category based on the number rather than the anatomical location of metastatic lymph nodes (LNs) in the 7th edition (3, 4). Briefly, patients with 1–3 positive LNs were classified into the N1 category and those with ≥4 positive LNs were classified into the N2 category. To ensure accurate staging, the AJCC cancer manual recommends examining at least six LNs. However, the number of LNs retrieved was usually less than 6 in most literatures. A multi-institutional study including 214 GBC patients reported that the median number of LNs examined was 4 (5). Skye et al. showed that 28.2% of 2,955 GBC patients had more than one LN retrieved based on the Surveillance, Epidemiology, and End Results (SEER) between 1991 and 2005 (6). Even after 2010, the mean number of LNs examined was only 4.028 (7). Therefore, there is an urgent need for an alternative index to accurately classify the LN status in case fewer than six LNs are retrieved.

Lymph node ratio (LNR), the ratio between the number of cancer positive LNs and the total retrieved surgically LNs, is a common prognostic indicator for various malignant tumors including pancreatic, colorectal, gastric, lung, and prostate cancer (8–12). Choi BG et al. (13) reported that LNR is an independent prognostic factor for GBC patients undergoing curative surgery. However, it is not clear whether LNR can substitute for LN count in the AJCC staging system, especially for GBC patients with fewer than six LNs retrieved. To this end, we evaluated the possibility of incorporating LNR into the AJCC staging system using data from the SEER database.

## Materials and Methods

### Ethics Statement

This study was approved by the institutional review board of First Affiliated Hospital of Jiamusi University. Patients from the SEER database had previously consented to participate in any scientific research worldwide.

### Patients

The data of GBC patients entered into the SEER database between 2004 and 2013 was included based on the following criteria: (1) age ≥ 18 years, (2) positive histological diagnosis, (3) first primary tumor, (4) underwent curative surgery, (5) lack of radiotherapy before surgery, (6) no distant metastasis, (7) at least one resected LN, and (8) definite T category according to the AJCC TNM staging system (8th edition). Patients with unavailable follow-up information were excluded. All patient data were fully anonymized.

### Statistical Analysis

Data of demographic and clinico-pathological characteristics, including age, sex, race, grade, TNM stage, histological type, and the number of LNs examined, were collected. The overall survival (OS) was calculated from the date of initial diagnosis to death or last follow-up time and analyzed by Kaplan–Meier method. The optimal cutoff value of LNR was determined using X-tile software (Yale University, Version 3.6.1). The discriminatory power of staging system was evaluated by concordance index (CI). Statistical analyses were performed using SPSS version 20 and a two-tailed *p* < 0.05 was considered statistically significant.

## Results

### Baseline Characteristics

A total of 1,723 GBC patients were enrolled, including 226, 443, 924, and 130 cases with stage I, II, III, and IV, respectively, (Table 1). Patients with fewer than six LNs examined accounted for the majority of cases (1,353, 78.5%). The median age of entire cohort was 68 years (21 to 96), and 1,207 (70.1%) patients were female. A higher proportion of patients (58.3%) had well- or moderately differentiated tumors (grade I + II) compared to poorly differentiated or undifferentiated tumors (grade III + IV), and adenocarcinoma was the most common histological type. The mean number of examined LNs was 3.8 in the entire cohort, and, respectively, 1.9 and 10.7 in patients with <6 or ≥6 LNs.

**TABLE 1:** Demographic and tumor characteristics for entire cohort.

Characteristics	All (%)	Patients with <6 LNs examined (%)	Patients with ≥6 LNs examined (%)	*P* value
Total No.	1723	1353	370	
Age, years				<0.001
Median (range)	68 (21–96)	69 (22–96)	65 (21–91)	
Sex				0.879
Male	516 (29.9%)	404 (29.9%)	112 (30.3%)	
Female	1207 (70.1%)	949 (70.1%)	258 (69.7%)	
Race				0.762
White	1338 (77.7%)	1049 (77.5%)	289 (78.1%)	
Black	191 (11.1%)	148 (11.0%)	43 (11.6%)	
Others	194 (11.2%)	156 (11.5%)	38 (10.3%)	
Grade				0.533
I + II	1004 (58.3%)	779 (57.6%)	225 (60.8%)	
III + IV	603 (35.0%)	481 (35.6%)	122 (33.0%)	
Unknown	116 (6.7%)	93 (6.8%)	23 (6.2%)	
Stage				<0.001
I	226 (13.1%)	193 (14.3%)	33 (8.9%)	
II	443 (25.7%)	341 (25.2%)	102 (27.6%)	
III	924 (53.6%)	758 (56.0%)	166 (44.9%)	
IV	130 (7.6%)	61 (4.5%)	69 (18.6%)	
Histological type				0.763
Adenocarcinoma	1576 (91.5%)	1239 (91.6%)	337 (91.1%)	
Other*	147 (8.5%)	114 (8.4%)	33 (8.9%)	
No. of LNs examined				<0.001
Mean	3.8	1.9	10.7	

*Including cystic, mucinous and serous neoplasms, and epithelial neoplasms, etc.

Multivariable analysis of OS in patients with GBC showed that age, gender, grade, stage, and the number of LNs examined were independent prognostic factors for patients with inadequate LN examination (Table 2). Independent prognostic factors for patients with adequate LN examination included age, stage, histology, and the number of LNs examined. The number of LNs examined was the common prognostic factor. Among patients with inadequate LN examination, patients with 1 LN examined accounted for the highest proportion while those with 5 LNs examined accounted for the smallest proportion (Table 3). Furthermore, patients in the T2 and T3 categories had a higher number of LNs examined.

**TABLE 2:** Multivariable analysis of OS in patients with GBC.

Characteristics	Patients with <6 LNs examined		Patients with ≥6 LNs examined	
	HR (95% CI*)	*P* value	HR (95% CI*)	*P* value
Age, years	1.027 (1.021–1.034)	<0.001	1.015 (1.000–1.030)	0.044
**Sex**				
Male	Reference		Reference	
Female	0.839 (0.718–0.980)	0.026	0.828 (0.590–1.161)	0.273
**Race**				
White	Reference		Reference	
Black	1.193 (0.952–1.495)	0.126	1.176 (0.707–1.958)	0.532
Others	1.051 (0.826–1.338)	0.685	0.880 (0.504–1.537)	0.653
**Grade**				
I + II	Reference		Reference	
III + IV	1.283 (1.101–1.495)	0.001	1.132 (0.815–1.571)	0.460
Unknown	1.063 (0.781–1.445)	0.699	0.839 (0.402–1.752)	0.641
**Stage**				
I	Reference		Reference	
II	1.021 (0.756–1.380)	0.891	1.598 (0.548–4.657)	0.391
III	3.104 (2.401–4.014)	<0.001	5.475 (1.976–15.174)	5.475
IV	6.747 (4.610–9.875)	<0.001	10.895 (3.857–30.775)	<0.001
**Histological type**				
Adenocarcinoma	Reference		Reference	
Other	0.952 (0.763–1.189)	0.666	1.693 (1.089–2.634)	0.019
No. of LNs examined	0.848 (0.794–0.906)	<0.001	1.033 (1.005–1.061)	0.020

*Confidence interval.

**TABLE 3:** Patient distributions stratified by the number of LNs examined.

	<6 LNs (%)	1 LN (%)	2 LNs (%)	3 LNs (%)	4 LNs (%)	5 LNs (%)	6 or More LNs (%)
Total	1353	759	246	162	106	80	370
T1	230 (17.0%)	145 (19.1%)	41 (16.7%)	25 (15.4%)	9 (8.5%)	10 (12.5%)	40 (10.8%)
T2	610 (45.1%)	338 (44.5%)	122 (49.6%)	63 (38.9%)	52 (49.1%)	35 (43.8%)	177 (47.8%)
T3	468 (34.6%)	257 (33.9%)	71 (28.8%)	66 (40.7%)	41 (38.7%)	33 (41.2%)	134 (36.2%)
T4	45 (3.3%)	19 (2.5%)	12 (4.9%)	8 (5.0%)	4 (3.7%)	2 (2.5%)	19 (5.2%)

### LNR and TNrM Staging

**FIGURE 1:**
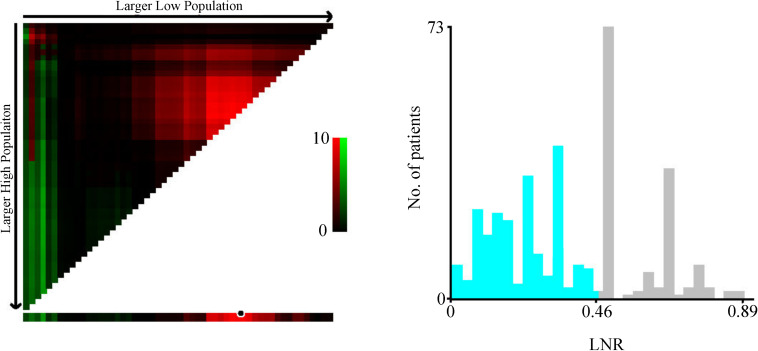
Optimal cutoff value produced by X-tile software.

**FIGURE 2:**
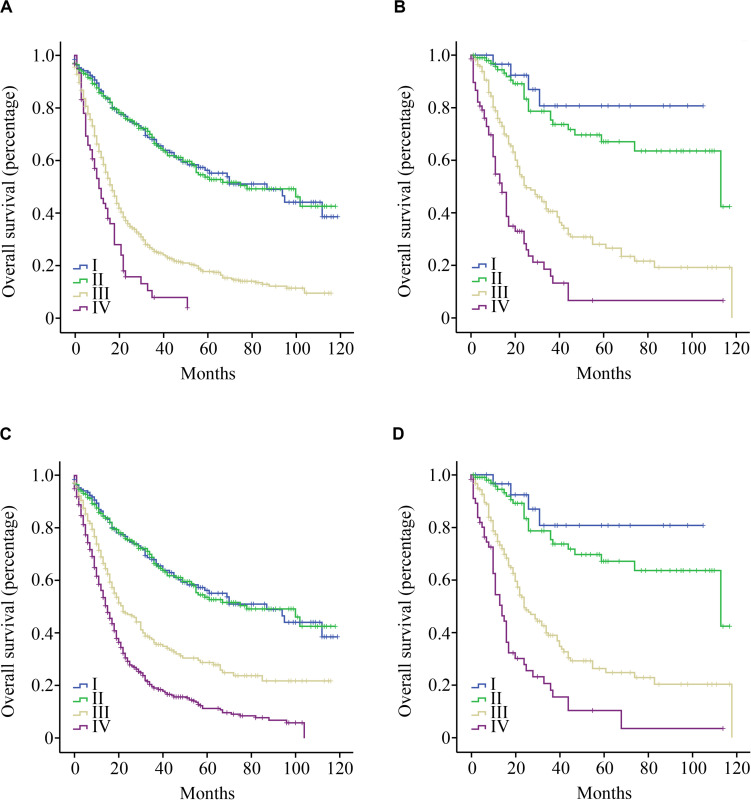
Overall survival curves for patients with GBC by LN count. **(A)** Patients with fewer than six LNs examined according to the TNM staging system; **(B)** Patients with six or more LNs examined according to the TNM staging system; **(C)** Patients with fewer than six LNs examined according to the TNrM staging system; and **(D)** Patients with six or more LNs examined according to the TNrM staging system.

The optimal cutoff value of LNR was 0.46 (Figure 1). The hazard ratio (HR) of patients with LNR above 0.46 was 1.29 (patients with LNR lower than 0.46 were the reference). As shown in the survival curves in Figure 2, the latest AJCC TNM staging system stratified patients with six or more LNs examined with greater accuracy compared to those with fewer than six LNs (CI, 0.704 vs. 0.636, Figures 2A,B). We then incorporated LNR into the TNM staging system in place of the N category (TNrM staging system). The TNrM demonstrated a stronger discriminatory power compared to TNM in patients with fewer than six LNs (CI, 0.649 vs. 0.636, Figure 2C). For patients with six or more LNs examined, TNrM and TNM showed similar discriminatory powers (CI, 0.694 vs. 0.704, Figure 2D). Additionally, those patients with only one LN examined could only have 0 or 1 LN positive, which might bring an element of bias. Therefore, we excluded the patients with only one LN harvested and re-analyzed these data. For patients with fewer than six LNs examined, the CI of TNrM staging system was 0.651, which was still higher than 0.636 (Supplementary Figure 1).

**FIGURE 3:**
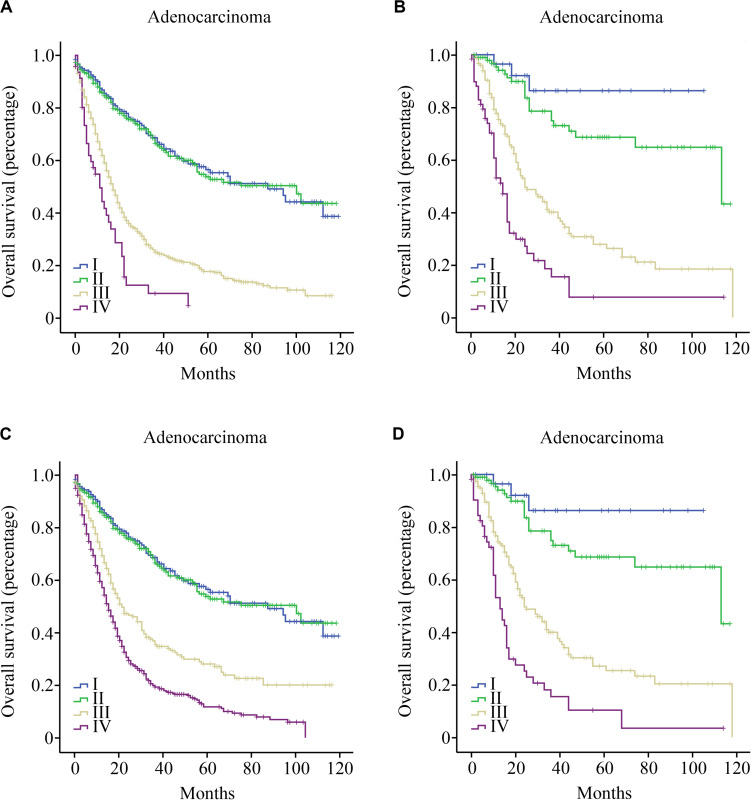
Overall survival curves for patients with gallbladder adenocarcinoma by LN count; **(A)** Patients with fewer than six LNs examined according to the TNM staging system; **(B)** Patients with six or more LNs examined according to the TNM staging system; **(C)** Patients with fewer than six LNs examined according to the TNrM staging system; and **(D)** Patients with six or more LNs examined according to the TNrM staging system.

Gallbladder adenocarcinoma (GBA), the most common histological type of GBC, was assessed separately. Similarly, TNM stratified patients with six or more LNs examined with greater accuracy compared to those with fewer than six LNs (CI, 0.706 vs. 0.638, Figures 3A,B). TNrM demonstrated a stronger discriminatory power compared to TNM in patients with fewer than six LNs examined (CI, 0.651 vs. 0.638, Figure 3C). For patients with six or more LNs examined, TNrM and TNM showed similar discriminatory powers (CI, 0.704 vs. 0.706, Figure 3D).

## Discussion

The AJCC TNM staging system for GBC has been continually updated every 7 or 8 years. In the 6th edition (2002), the N category was determined in terms of the presence or absence of LN metastasis (N0—no LN metastasis; N1—LN metastasis) (14). In the 7th edition (2010), LN metastases were further divided into N1 and N2 levels depending on their anatomical location. In the latest edition, the number of positive LNs rather than location is the defining factor of the N category. However, the number of LNs examined had not been given much credence in the previous clinical guidelines. We found that more than three-quarters of GBC patients had fewer than six LNs resected. Therefore, the 8th edition of AJCC TNM staging system cannot accurately stratify most GBC patients by death risk. In the present study, we introduced an alternative index LNR for the prognostic prediction of GBC patients with fewer than six LNs examined, and we can complement the TNM staging system for better management and surveillance. Additionally, the mean LN number of patients with adequate LN examination was up to 10.7 (much higher than 1.9 of patients with inadequate LN examination), which may result from high proportion of patients with advanced disease (Table 1). Admittedly, with the application of the 8th TNM staging system, the number of specimens with adequate LN examination will increase over time.

Stage III patients (758 cases) with fewer than 6 LNs examined in the TNM staging system were reclassified into stage III (248 cases) and stage IV (510 cases) in the TNrM staging system. The median OS for 248 cases of stage III was 22 months while 510 cases of stage IV was 15 months (*p* < 0.001). For stage IV patients with 6 and more LNs examined in the TNM staging system, the median OS was 14 months, which was similar to the 15 months (*p* = 0.489). Therefore, reclassification of stage III patients (758 cases) with fewer than 6 LNs examined in the TNM staging system was appropriate and could give more accurate data for prognostication.

Other staging systems for GBC, including LNR, have been proposed as more accurate staging methods. Amini N et al. (5) indicated that LNR performed better than LN location when used for GBC diagnosis, especially for patients with four or more LNs examined. Similarly, Kim SH et al. (15) found that the number of positive LNs had a positive effect on prognostic performance compared to LNR in T3 GBC when the number of LNs examined was eight or more. These studies were conducted on small cohorts and did not compare the prognostic value of the indices when the number of LNs examined was fewer than six. Ito H et al. (16) showed that a minimum of six LNs was required for accurate staging, although 70.0% (85/122) of their entire cohort had fewer than six LNs examined, which was similar to our results. They did not either mention how to stratify patients with fewer than six LNs. To our best knowledge, we have developed an alternative staging system for the first time to stratify GBC patients with fewer than six LNs retrieved.

There were some limitations in our study that ought to be addressed. Even in the SEER database, one of the largest clinical database representing approximately 28% of the US population (17), it includes only 370 cases with six or more LNs. The 8th AJCC TNM staging system needs to be validated for GBC using more samples. Second, recurrence-free survival was not recorded in the SEER database and the relevant analysis cannot be performed. Third, data from the SEER database were retrospective, which could result in bias to some extent. Prospective studies need to be performed to verify our staging system.

## Data Availability Statement

Publicly available datasets were analyzed in this study. This data can be found here: https://seer.cancer.gov/data/.

## Ethics Statement

This study was approved by the institutional review board of First Affiliated Hospital of Jiamusi University.

## Author Contributions

JL, YR, WJ, and DF made substantial contributions to the design of the study, carried out the analysis, and interpreted the data. BZ and CT contributed to the review of previous literature. JL and YS contributed substantially to the data discussion and critically commented on the manuscript for scientific content. All authors were responsible for the quality of the overall manuscript and approved the final version.

## Conflict of Interest

The authors declare that the research was conducted in the absence of any commercial or financial relationships that could be construed as a potential conflict of interest.
