# Supplementary figures and images for: Lymph Node Ratio-Based Staging System for Gallbladder Cancer With Fewer Than Six Lymph Nodes Examined
Source: Front Oncol. 2020 Sep 25;10:542005. doi: 10.3389/fonc.2020.542005 (PMC7546035; doi:10.3389/fonc.2020.542005)

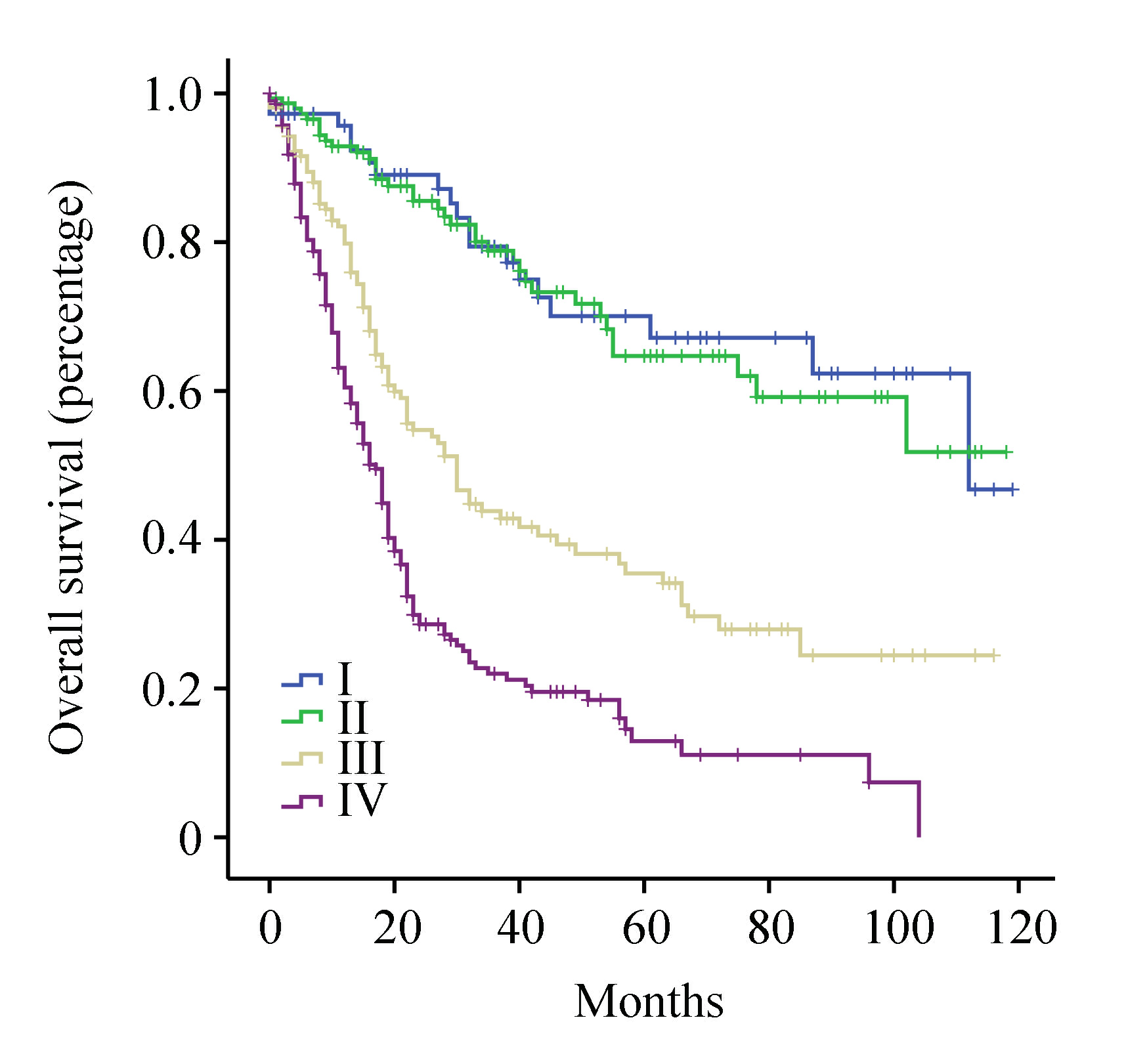

Supplement: Supplementary file 1 [file Image_1.tif]
